# Effect of Sensors on the Reliability and Control Performance of Power Circuits in the Web of Things (WoT)

**DOI:** 10.3390/s16091430

**Published:** 2016-09-06

**Authors:** Sungwoo Bae, Myungchin Kim

**Affiliations:** 1 Department of Electrical Engineering, Yeungnam University, Gyeongsan 38541, Korea; sbae@yu.ac.kr; 2 Agency for Defense Development, Daejeon 34186, Korea

**Keywords:** power circuit, Web of Things (WoT), energy harvesting, reliability, filter, sensors

## Abstract

In order to realize a true WoT environment, a reliable power circuit is required to ensure interconnections among a range of WoT devices. This paper presents research on sensors and their effects on the reliability and response characteristics of power circuits in WoT devices. The presented research can be used in various power circuit applications, such as energy harvesting interfaces, photovoltaic systems, and battery management systems for the WoT devices. As power circuits rely on the feedback from voltage/current sensors, the system performance is likely to be affected by the sensor failure rates, sensor dynamic characteristics, and their interface circuits. This study investigated how the operational availability of the power circuits is affected by the sensor failure rates by performing a quantitative reliability analysis. In the analysis process, this paper also includes the effects of various reconstruction and estimation techniques used in power processing circuits (e.g., energy harvesting circuits and photovoltaic systems). This paper also reports how the transient control performance of power circuits is affected by sensor interface circuits. With the frequency domain stability analysis and circuit simulation, it was verified that the interface circuit dynamics may affect the transient response characteristics of power circuits. The verification results in this paper showed that the reliability and control performance of the power circuits can be affected by the sensor types, fault tolerant approaches against sensor failures, and the response characteristics of the sensor interfaces. The analysis results were also verified by experiments using a power circuit prototype.

## 1. Introduction

**Figure 1:**
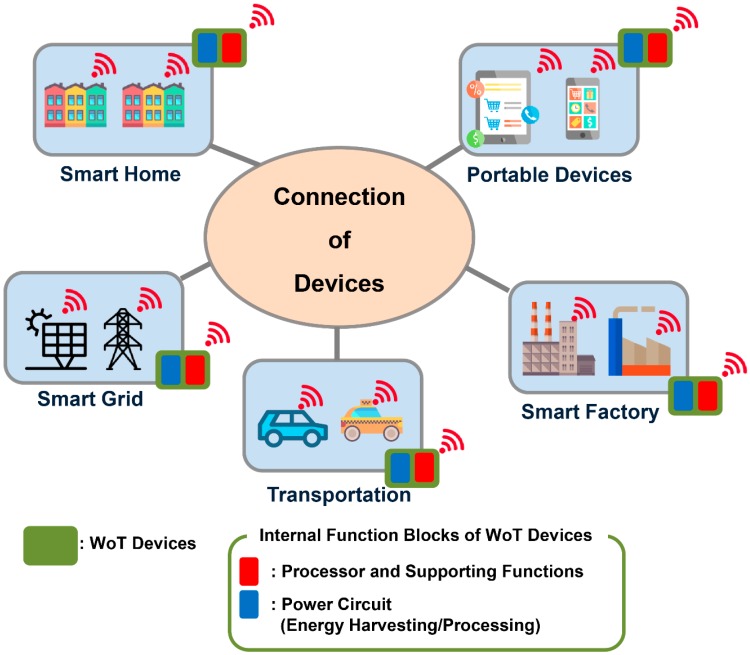
Role of power circuits in the WoT.

The use of advanced power processing circuit technologies has increased continuously for numerous applications such as wireless sensor networks [1,2], IoT devices [3,4], and home appliances [5]. To realize a genuine Web of Things (WoT) environment, a reliable power circuit for various connected devices is required [6]. As shown in Figure 1, the power circuits inside such devices perform energy harvesting and power processing so that the devices are operational and connected to the WoT. The processors and transmitters of such WoT devices can successfully function using the energy that is harvested and processed by the power circuits. Since power circuits and different types of power sources are commonly used in the WoT devices [7,8], their design engineers should not overlook the effect of power circuits (e.g., energy harvesting interfaces) on system performance factors such as reliability [9], cost [10], and efficiency [11]. Examples of power sources for WoT applications that are considered in this paper include batteries [2] and solar cells [1,7]. To enable the effective and efficient operation of different power sources, the system designers should consider the reliable circuit control strategies and power management algorithms for power circuits used in WoT devices.

**Figure 2:**
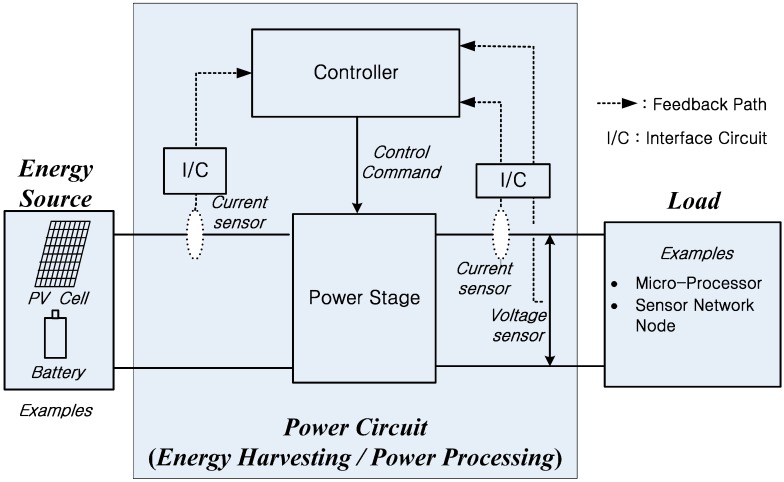
Block diagram of a typical power conversion approach for a WoT connected device.

Figure 2 shows a block diagram of a typical power circuit configuration for a WoT-connected device. While the actual power conversion in the power circuit is performed by the power stage, controllers are generally used to control the major variables of interest, such as voltage and current. Since sensors are typically used for feedback in control systems, satisfactory operation of the controller would not be possible when the feedback information is unavailable, which may result from the failure of sensors. As shown in Figure 2, an interface circuit exists between the sensor output and the control input. Hence, the characteristics of the actual feedback signal are expected to be determined not only by the sensors, but also by their interface circuits. In other words, the failure rate of sensors can affect the reliability of power conversion circuits [12]. In the same manner, the dynamics of the sensors with their interface circuits can affect the control performance [13]. As a power circuit control method generally requires the installation of sensors, this paper reports the effects of sensors and their interface circuits on the performance of power circuits. In particular, this study focuses on how the reliability and control performance are affected by the sensor failure rates and dynamics.

The analysis process, observation, and verification results of this study address hardware and system design challenges involving the connection of various WoT devices. Specifically, the contributions of this study can be summarized as follows:

First, this research provides a comprehensive analysis result on the reliability and control performance of WoT devices by considering the effects of sensors and their interface circuits. Although the sensor reliability has been considered explicitly in several case studies [12,14], the use of sensor reliability data was limited to only investigating the detailed failure modes and failure criticality. Compared to previous research, this study does not only focus on the reliability of the sensor, but also examines how alternative sensing mechanisms other than the direct feedback from sensors can improve the system reliability. For example, the use of current information reconstruction or estimation schemes that can increase the fault tolerance against current sensor faults is considered as a method to increase the system reliability. While research works on the reconstruction or estimation of voltage/current feedback have been performed [15,16,17,18,19], this paper provides the quantitative analysis results on the level of system reliability improvement that can be achieved.

Second, this paper considers the practical hardware design aspects of power circuits in sensor interfaces that have not been explored thoroughly in the past. In particular, this study is motivated by the following observations: (1) measurement sensors used in the power circuits can also fail or wear out [12]; and (2) interface circuits are generally used with sensors to address issues related to electromagnetic interference (EMI), analog-to-digital conversion (ADC), and switching mode power regulation [2]. Through both theoretic analysis and circuit oriented verification, this paper demonstrates that such practical constraints can affect the reliability and regulation performance of power circuits that are commonly used for energy harvesting in WoT devices.

Third, a system designer can easily apply the analysis approach of this paper to study the effects of different circuit configurations and feedback schemes that are used in power circuits for different types of embedded devices. When multiple approaches are used to acquire feedback of the same variable, the reliability analysis framework considered in this paper can be applied easily to reliability calculation models of such approaches. As the considered reliability analysis method provides quantitative results, the system designer can perform a more comprehensive and practical trade-off study among the various design alternatives during the system planning stage. These results can also be used as a metric to perform design optimization to meet various objectives (e.g., cost, reliability, and complexity). For example, energy harvesting interfaces of sensors can be optimized so that the design approach with the highest reliability level can be selected among various design candidates. While research works on designing sustainable energy harvesting approaches for sensors have received continuous interest [1,7,8,20], most previous research works have focused only on the development of circuit and power control approaches; and there seem to be limited studies on performing a system level analysis considering the effect of power circuits on the reliability characteristics.

The remainder of this manuscript is organized as follows: the following section reviews the related prior research works. Section 3 discusses the effects of sensors on the system reliability and introduces the reliability analysis approach. The reliability analysis result is also provided. Section 4 shows how the dynamics of the sensor interface circuit affect the system control performance. The verification results of both simulation and experiments are provided in Section 5. Finally, Section 6 concludes the paper by summarizing the findings and introducing directions for future work.

## 2. Review of Related Works

The design of power circuits in the WoT is critical to enabling sustainable operation of devices that are powered by batteries [2] and PV cells [1,7]. Therefore, reliability of the power circuits should be high enough to maximize the operational period and life cycles of WoT devices. Recently, research works on a quantitative reliability analysis and the fault tolerant operation of power circuits have been performed actively to establish a systematic approach for a reliable system design. For example, the effects of different switching device configurations (i.e., discrete power switches and integrated power modules) on the system reliability were studied [21]. Comparison results in [21] showed that the overall reliability performance can differ depending on how power switches are configured even in the same circuit topology. A design approach for power circuits in aerospace applications, which requires a high level of reliability, was explored [9]. In particular, the effects of different capacitor types, pulse-width-modulation (PWM) strategies, switching frequencies, and power switch configurations on the system reliability were studied based on the reliability prediction results. The effects of different circuit topologies on the reliability of PV system interfaces were also studied [22]. Studies on reliability calculation frameworks have been proposed for actuation systems and renewable power systems [12,23,24]. To design a system with improved reliability, a control approach for power circuits with hardware redundancy was also introduced [25]. Previous studies [14,25,26] not only provided reliability analysis results on the case studies of control approaches that have a redundancy, but also discussed how practical fault scenarios can be handled by the proposed control approach. Although reliability studies on power circuits have been investigated in previous studies, the majority of these studies have focused only on power stage components, such as the power devices and reactive components. In other words, the effects of sensors on the system reliability have not been fully examined in power circuits used in WoT devices. In order to address such gap in the literature, this paper performed a reliability analysis of power circuits with a focus on the effects of sensors and alternative sensing approaches.

Studies on the approaches that can minimize the use of sensors have also been performed with motivations to realize cost effective power management solutions and highly integrated power circuits in a compact size. For example, a current estimation approach that uses an Extended Kalman Filter (EKF) has been used in a boost converter [15]. Approaches that use either inductor voltage [16,17] or inductor voltage-second [18] information to estimate the inductor current have been explored to sense the inductor current without sensors. Using the approach of [19], it is possible to know the current information of each individual stage in an interleaved dc converter by installing only a single sensor at the common dc link instead of installing sensors at each stage. These approaches can contribute to decreasing the overall cost and size of the power circuit by reducing the number of sensors required. From a reliability perspective, such reconstruction or estimation techniques can be considered for designing power circuit interfaces with fault tolerant capability [27]. Not only does the power processing circuit become less constrained to the status of the sensor, but these estimation techniques can also be used as a back-up sensing mechanism that can be activated as soon as a fault in the sensor is detected. Although the approaches of previous studies can be applied to design a power processing circuit that is tolerant to sensor faults, a quantitative analysis of reliability improvement should also be required for optimized approaches that can satisfy both the cost and reliability requirements.

Previous studies have also shown that the control performance can be affected by the interface circuits that are commonly used with sensors. For example, [13] demonstrated through a series of experiments that subharmonic stability problems can arise in buck converters depending on the parameters of the interface circuit. The effects of the current sensor or its interface circuit on the stability performance of current mode controlled power converters have also been reported [28].

## 3. Reliability of Sensors and Systems

This section introduces the power circuit configuration, control approach and reliability model that are considered in the following discussion of this paper.

### 3.1. Control and Reliability of Power Circuits

**Figure 3:**
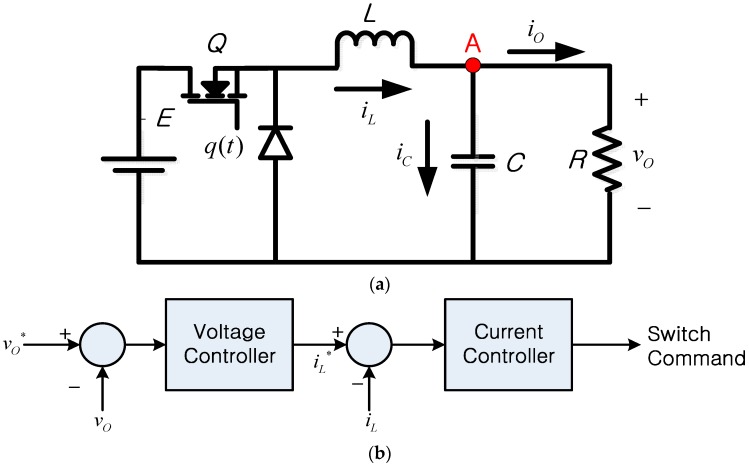
(**a**) Buck converter configuration; (**b**) control block diagram.

This paper considers two representative power processing circuit configurations. Figure 3 shows the configuration and control approach of a dc-dc buck converter. Thanks to its ability to step down the output voltage, a buck converter is commonly used for energy harvesting in low power applications, such as wireless sensor networks [29] and small portable devices [30]. The buck converter can be modeled as follows [31]:

(1) $$L\frac{di_{L}}{dt}=q(t)E-v_{o}$$

(2) $$C\frac{dv_{o}}{dt}=i_{L}-\frac{v_{o}}{R}$$

where $i_{L}$ is the inductor current, E is the input (source) voltage, $v_{o}$ is the capacitor (load) voltage, *L* is the inductance, *C* is the capacitance, *R* is the load resistance, and *q*(*t*) is the switching function of the power switch *Q*. The switching function *q*(*t*) is defined as:

(3) $$q(t)=\{\begin{array}{l} 1,\;\mathrm{when}\;Q\;\mathrm{is}\;\mathrm{on}\\ 0,\;\mathrm{when}\;Q\;\mathrm{is}\;\mathrm{off} \end{array}$$

**Figure 4:**
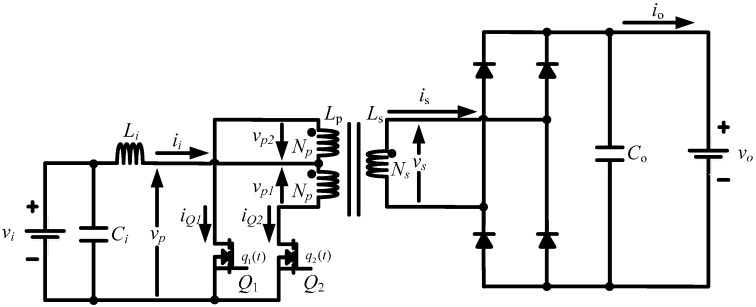
Configuration of a current-fed push-pull converter.

A current-fed push-pull interface can be considered when a boost operation through isolation at higher power levels is required for safety. Figure 4 shows the circuit configuration of this boost power processing unit, and the circuit can be modeled as [32]:

(4) $$L_{i}\frac{di_{i}}{dt}=v_{i}-v_{p}$$

(5) $$C_{o}\frac{dv_{o}}{dt}=\left|i_{s}\right|-i_{o}$$

(6) $$\frac{d\phi }{dt}=\frac{v_{s}}{N_{s}}=\frac{v_{p1}}{N_{p}}=-\frac{v_{p2}}{N_{p}}$$

(7) $$v_{p}=\left[1-q_{1}(t)\cdot q_{2}\left(t\right)\right]\cdot \frac{N_{p}}{N_{s}}\cdot v_{o}$$

(8) $$\phi =\frac{L_{p}}{N_{p}}\left(i_{Q1}-i_{Q2}\right)-\frac{L_{s}}{N_{s}}i_{s}$$

(9) $$v_{s}=\left[q_{1}(t)-q_{2}(t)\right]\cdot v_{o}$$

where *i_i_* is the current of inductor *L_i_*; *v_i_* is the input voltage; *v_p_* is the primary voltage; *v_o_* is the capacitor (output) voltage; *C_o_* is the output capacitance; *i_s_* is the secondary current of the transformer; *i_o_* is the output current; ϕ is the transformer magnetization flux; *v_s_* is the transformer secondary voltage; *N_s_* is the number of turns in the transformer secondary side; *N_p_* is the number of turns in the transformer primary side; and *q_i_*(*t*) is the switching function of the power switch *Q_i_* (*i* = 1, 2). The switching function *q_i_*(*t*) is defined in the same manner as Equation (3).

To regulate the output voltage, *v_o_*, a cascaded control approach [33] (i.e., Figure 3b) was considered. As shown in Figure 3b, the outer voltage controller generates the reference command for the inner current controller, and the current controller generates the control command for the power switch, *Q*. To regulate the load voltage, the voltage controller requires the feedback of the load voltage value, and the current controller uses the feedback of the inductor current through sensors [34,35,36]. Therefore, failure to provide the feedback of accurate information on the inductor current to the current controller might cause an unsatisfactory performance or failure in the voltage regulation of the power circuit. This paper uses the inductor current for voltage regulation purposes, whereas the current information has been used in other applications of power circuits for various objectives. A representative example of energy harvesting purposes is the maximum power point tracking (MPPT) method of PV modules [7].

While the control performance is determined by the controller configuration, controller gains, and the circuit parameter values, the reliability performance is determined by the failure rate of the interface hardware. In this paper, it is assumed that the reliability of each hardware component can be modeled to have an exponential distribution with a constant failure rate (λ), as in previous studies [9,10,12,25]. That is, the reliability function of a component (i.e., *R*(*t*)) is expressed as [9,10,22]:

(10) $$R(t)=e^{-\lambda t}$$

where λ is the failure rate (failures/hours), and *t* is time (hours).

### 3.2. Reliability Analysis Considering Sensors

Although each hardware device of the power circuit (e.g., capacitor, inductor, and power switch) can fail during the system operation, this study simplifies the reliability analysis by assuming that only the current sensors are subject to failures. Reliability studies that consider the failures of other components can be found in other studies [22,24]. By assuming that only the inductor current sensor can fail, the probability that the power circuit is operational (i.e., *R_PC_*(*t*)) can be calculated by using the failure rate data of the current sensor as follows:

(11) $$R_{PC}\left(t\right)=R_{CS}\left(t\right)=e^{-\lambda_{CS}t}$$

where *R_CS_*(*t*) is the reliability function of the current sensor, and *λ_CS_* is the failure rate of the current sensor. Examples of current sensor failure rate values can be found in previous reliability analyses [14,25] or in datasheets provided by the manufacturers [37].

While the reliability can be calculated using Equation (11) based on the assumption that the inductor current sensor is the only method for sensing the inductor current value, the inductor current can still be known without requiring explicit feedback information of the sensor output. For example, the inductor current can be estimated using the inductor voltage value as follows [16,17]:

(12) $$i_{L}=\frac{1}{L}\int v_{L}dt=\frac{1}{L}\int \left(q\left(t\right)E-v_{o}\right)dt$$

where *v_L_* is the inductor voltage. This estimation approach is possible based on the voltage-current relationship of the inductors. When the inductor current feedback is performed based on an alternative method instead of using the current sensor, the failure rate to be considered in Equation (11) should be modified to represent the actual sensing configuration. For example, the failure rate in Equation (11) should represent the properties of the voltage sensor instead of the current sensor if the inductor voltage based estimation approach (i.e., Equation (12)) is used. A different approach for estimating the inductor current is by reconstructing the inductor current value through other current information of the interface. In particular, the capacitor current and load current can be used for such purposes. Furthermore, such alternative sensing approaches can be used as a backup mechanism with an objective to achieving fault tolerant capability against the faults of the inductor current sensor. The system can be designed such that the controller receives feedback from the inductor current sensor as primary, and the source of the inductor current value feedback can be switched from the current sensor to an alternative approach (e.g., Equation (12)) when a fault in the inductor current sensor is detected.

When such alternative approaches are available, a more comprehensive analysis can be performed to quantify the level of reliability improvement. Table 1 lists the possible operation conditions of the three current sensors in the buck converter.

Cases 1 to 4 represent the operation scenarios that the inductor current information is available through the inductor current sensor itself. Since the explicit feedback information of an inductor current value from the sensor is available, the system is regarded as being operational regardless of the operation status of the capacitor current sensor and the load current sensor. In other words, the system is operational as long as the inductor current sensor is healthy. Hence, the probability (or the system reliability function, *R_PC1_*) that the system is operational can be expressed as [10]:

(13) $$R_{PC1}=p_{IL}=e^{-\lambda_{CS}t}$$

where *p_IL_* is the probability that the inductor current sensor is healthy.

When the inductor current sensor is not operational, as in the other cases (i.e., Cases 5 to 8), voltage regulation can still be performed using alternative approaches for current feedback information. A detailed explanation of these cases is as follows.

In the case that the capacitor current sensor and the load current sensor are operational (i.e., Case 5), the instantaneous inductor current value can be reconstructed from the output of the capacitor current sensor and the output of the load current sensor as follows:

(14) $$i_{L\_rec}=i_{C}+i_{O}$$

where *i_L_rec_* is the reconstructed inductor current, *i_C_* is the measured capacitor current, and *i_O_* is the measured load current. Applying Kirchhoff’s current law at node A of Figure 3a enables such a current reconstruction approach. Therefore, by installing a capacitor current sensor, a load current sensor, and a mechanism (e.g., algorithm) that can provide the result of Equation (14) to the current controller, the reliability of the system (i.e., *R_PC2_*) can be increased from Equations (13) to (15) as follows:

(15) $$R_{PC2}=R_{PC1}+\Delta R_{1}=R_{PC1}+q_{IL}\cdot p_{IC}\cdot p_{IO}$$

where *q_IL_* is the probability that the inductor current sensor fails (*q_IL_* = 1 − *p_IL_*), *p_IC_* is the probability that the capacitor current sensor is operational, and *p_IO_* is the probability that the load current sensor is operational. An increase in reliability, $\Delta R_{1}$, is achieved at the expense of installing additional current sensors (i.e., capacitor current sensor and load current sensor) and implementing the reconstruction approach, as expressed in Equation (14). Depending on the level of reliability required, some system design cases will require such a backup scheme at the cost of installing additional sensors.

Although only the capacitor current sensor is available as shown in Case 7, it is still possible to estimate the load current under the assumption that the load resistance is known. As the output voltage sensor would have already been installed to perform voltage regulation in the buck converter, the load current estimation can be performed using the sensed output voltage without installing extra sensors. The inductor current value can be estimated using the capacitor current and the output voltage value as follows:

(16) $$i_{L\_est}=i_{C}+i_{O\_est}=i_{C}+\frac{v_{O}}{R}$$

where *i_O_est_* is the estimated load current. Accordingly, the reliability of the system (i.e., *R_PC3_*) can be increased further from Equations (15) to (17) as

(17) $$R_{PC3}=R_{PC2}+\Delta R_{2}=R_{PC2}+q_{IL}\cdot p_{IC}\cdot q_{IO}\cdot p_{VO}$$

where *q_IO_* is the probability that the load current sensor fails (*q_IO_* = 1 − *p_IO_*), and *p_VO_* is the reliability function of the output voltage sensor. The primary objective of this study is not to develop fault tolerant control or sensing approaches for power circuits but to introduce and perform a quantitative reliability analysis considering the effects of sensors and various alternative feedback approaches. Based on this objective, this paper considered rather straightforward sensing approaches for the reliability analysis.

**Figure 5:**
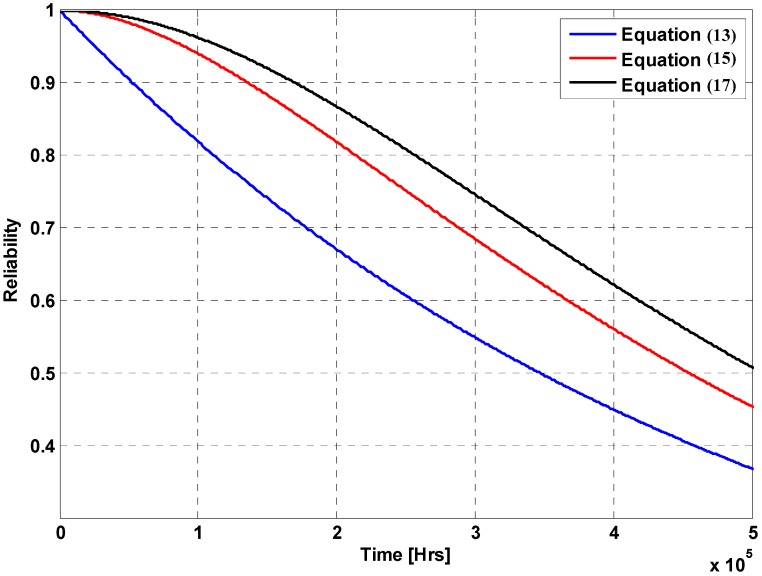
Reliability calculation results.

Figure 5 shows the reliability computation results from Equations (13), (15) and (17) using the sensor failure data [14]: λ = 2 failures/10^6^ hours. The reliability value shows a remarkable increase with increasing number of cases that make the system operational by applying additional mechanisms for current sensing. In other words, the system reliability increases with increasing number of alternative approaches for acquiring the current value. For example, the reliability values at *t* = 10,000 h for Equations (13), (15) and (17) are 0.9802, 0.9992 and 0.9996, respectively. The reliability analysis result of this study, such as the plot shown in Figure 5, can be used to characterize the reliability of power circuits in various WoT devices. In addition, the analysis result can also provide estimation on system availability of networks that consist of such WoT devices. Using the quantitative reliability data (i.e., Figure 5) from the proposed reliability analysis process, a power circuit designer can also choose proper sensors or alternative sensing approaches for a reliable power circuit in a more systematic and comprehensive way. As reliability performance can be considered during the design process, reliability issues of power circuits for energy harvesting and power processing in WoT devices can be addressed by applying the analysis approach of this study.

### 3.3. Further Considerations

As shown in Figure 5, the system reliability can be improved by considering alternative approaches that perform identical tasks (e.g., control and sensing). In the case of buck converter control, voltage regulation can also be performed using the cascaded control configuration that relies on the feedback of the capacitor current instead of the inductor current [38]. The capacitor current control approach can be activated when a fault in the original controller or in the inductor current sensor is detected so that the regulation performance is not interrupted by the resulting fault. With such back-up capability, Case 7 in Table 1 can also be included as an operational case without requiring a load current estimation and can contribute to improving the system reliability. In a similar manner, the effects of alternative approaches for estimating the current on the system reliability can be studied further. Instead of relatively simple approaches that were considered in the previous analysis, it is possible to include the effects of different types of advanced current reconstructions or estimation approaches that can be used when the inductor current sensor fails [39,40]. An estimation of the capacitor current can be considered, and even all current values can be estimated when the direct feedback from all current sensors is unavailable, as in Case 8. As the system is equipped with some level of fault tolerant capability by introducing redundancy in the control or sensing function, it is possible to expect some improvement in reliability similar to previous studies [14,25,26]. On the other hand, a reliability analysis that includes other alternative approaches was not performed in this paper because the development of alternatives is not in the scope of this paper. In addition, the actual applicability of these alternative approaches is decided not only by the reliability requirements, but also by the constraints of other factors, such as cost, complexity, and volume.

## 4. Dynamics of the Sensor Interface Circuit and System

**Figure 6:**
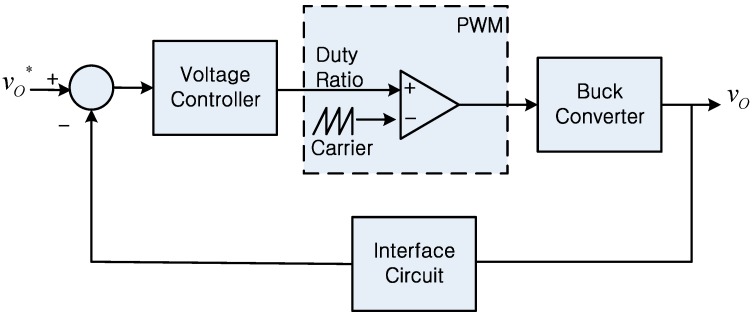
Control block diagram of a buck converter.

While Section 3 discussed how sensors and alternative sensing approaches can affect the reliability performance of the power circuits in WoT devices, this section discusses how sensors and their interface circuits could affect the control performance of power circuits. Analogous to the relationship between sensor failure rate and system reliability, the dynamic characteristics of the sensor itself and its interface circuit can also affect the control performance of power circuits. To illustrate such an effect, this paper considers the control block diagram of a buck converter whose output voltage is regulated through feedback control as shown in Figure 6 [31]. Since the actual feedback signal that is fed to the voltage controller is not the immediate sensor output but the signal that is processed by the sensor interface circuit, the signal characteristics of the voltage controller output (i.e., control command) would be affected by the properties of the interface circuit. Such interface circuits are commonly used to mitigate the effects of switching noises and to prevent aliasing [31,41,42].

To study the effects of interface circuits on the control performance, this paper examines the frequency response of the control loop gain [43] shown in Figure 6. In particular, this study is interested in exploring how the dynamics of the interface circuit affect the regulation performance when external disturbances in the load or the source are introduced. The loop gain of the control diagram shown in Figure 6 can be expressed as [43]:

(18) $$L_{c}\left(s\right)=G_{C}\left(s\right)\cdot G_{vd}\left(s\right)\cdot G_{IC}\left(s\right)$$

where *G_c_*(*s*) is the transfer function of the voltage controller, *G_vd_*(*s*) is the control input-to-output voltage transfer function of the buck converter, and *G_IC_*(*s*) is the transfer function of the sensor interface circuit. In feedback control systems, the loop gain has been used extensively for design and characterization purposes of their systems because the reference-to-output transfer function of the converter voltage in Figure 6 can be expressed as [43]:

(19) $$\frac{v\left(s\right)}{v^{*}\left(s\right)}=\frac{G_{C}\left(s\right)G_{vd}\left(s\right)}{1+G_{C}\left(s\right)G_{vd}\left(s\right)G_{IC}\left(s\right)}=\frac{G_{C}\left(s\right)G_{vd}\left(s\right)}{1+L_{c}\left(s\right)}$$

**Figure 7:**
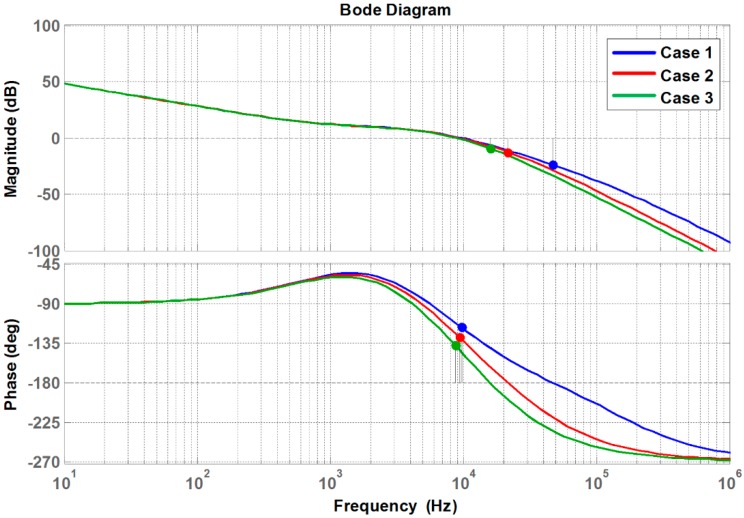
Bode plot of the loop gain with different sensor interface circuits.

Assuming that the voltage controller is a PI controller and the dynamics of the interface circuit are approximated as the first order low pass filter as:

(20) $$G_{IC}\left(s\right)=\frac{1}{\tau s+1}$$

where τ is the time constant of the interface circuit, the bode plot of the loop gain (i.e., Equation (18)) can be analyzed as shown in Figure 7 using the system parameter values listed in Table 2.

Figure 7 shows the effects of different time constants on the frequency response of the loop gain. Table 3 lists the values of the considered time constants. Although both the magnitude and the phase of the loop gain are affected by the change in the dynamics of the interface circuit (i.e., time constant), this paper focuses on the phase margin (PM) variation. In particular, the PM decreases with increasing time constant of the interface circuit. Considering that the performance of controlled systems is affected by the PM [42,43], it can be concluded that the control performance will be affected by the dynamics of the interface circuit. Such a decrease in the PM value will result in a greater oscillatory system response to disturbances. It is worth noting that a similar observation can be made for digital filters.

Although the dynamics of the interface circuit can be considered during the controller (e.g., control law and controller gains) design process, the characteristics of the interface circuits can be changed during the overall life cycle. For example, the capacitance or inductance values of the passive components in analog circuits can be affected by the operation conditions (e.g., temperature, humidity) or the aging of materials, and component tolerances [34,35]. When multiple sensing approaches are implemented, it is necessary to perform studies for all of the feedback options to ensure that the performance, particularly stability, is unaffected by the sensing mechanism. For digital controllers, in addition to the dynamics of the sensor interface circuit, the effects of analog-to-digital conversion (ADC), sampling, and computation can also be non-negligible [42,44]. The delay time caused by such digital tasks is dependent on the sampling frequency, complexity of the sensing approach, and the time required for fault detection or controller reconfiguration.

## 5. Results Analysis

**Figure 8:**
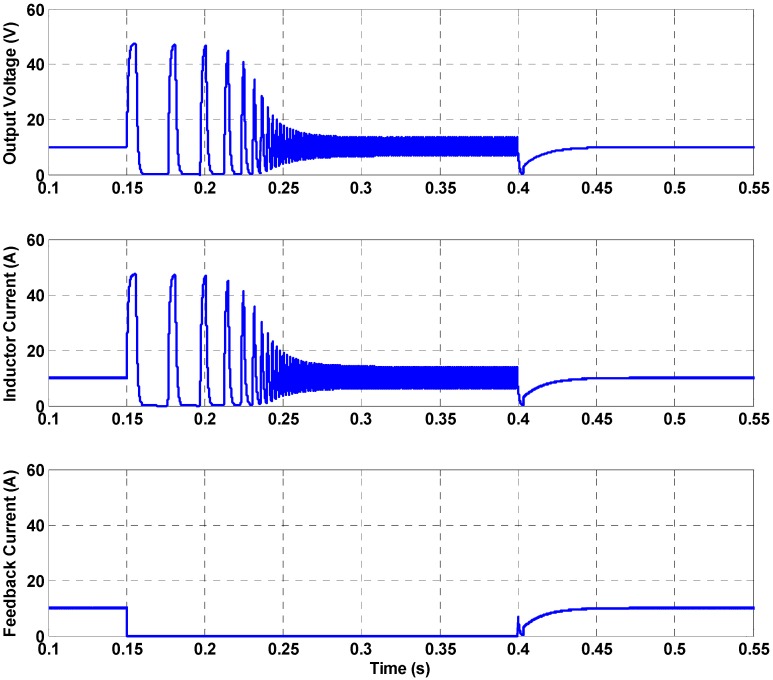
Effect of sensor faults on the control performance.

To demonstrate that the faults in the current sensor can be a direct reason for the failures of power circuits, a fault scenario was simulated for the buck converter using the parameters listed in Table 4 [33]. Figure 8 presents the effects of this sensor fault scenario on the control performance. The load voltage was regulated to 10 V using a cascaded control configuration as shown in Figure 3b. Initially, the buck converter shows an acceptable regulation performance owing to the controller, as shown in Figure 8. At *t* = 0.15 s, an inductor current sensor fault was introduced to the system by disconnecting the inductor current feedback loop as depicted in Figure 8.

A discrepancy between the actual inductor current value and the feedback current value exists in the simulation waveform between *t* = 0.15 s and *t* = 0.4 s because of the sensor fault. As soon as the fault is introduced to the current sensor, the load voltage becomes uncontrollable and the voltage fails to be regulated to the reference value, 10 V. Once the sensor fault is removed at *t* = 0.4 s and the current feedback is resumed, it can be seen that the load voltage is regulated to the reference value and the controllability is recovered. This result shows that a fault in the sensor can result in a failure of the power processing circuit interface. The cascaded control configuration (e.g., Figure 3b), which requires the feedback of both the voltage and current values, has been reported to improve control performance [31,33]. However, the results of Figure 8 show that the additional inner current control loop could cause system failure when a fault in the current sensor occurs. Such reliability characteristic is based on the fact that the cascaded control configuration generally requires feedback sensors for two control loops (i.e., the inner control loop and the outer control loop) to be available. In realistic settings, the protection logic of the converter would have been activated because of the excessive current level. Such activation would cause an interruption to the operation of the power circuit. Hence, sensor reliability performance should also be reviewed to ensure that the system reliability is not affected by the sensor failure rates. When a WoT device is required to have a relatively high reliability level, the alternative feedback approaches that were discussed in Section 3 can be used to achieve reliable power circuit operation that is more tolerant against current sensor faults. Among the various available approaches that could be considered as an alternative for acquiring feedback information, the reliability analysis results of Figure 5 can be used to select the most suitable approach from a system reliability perspective.

**Figure 9:**
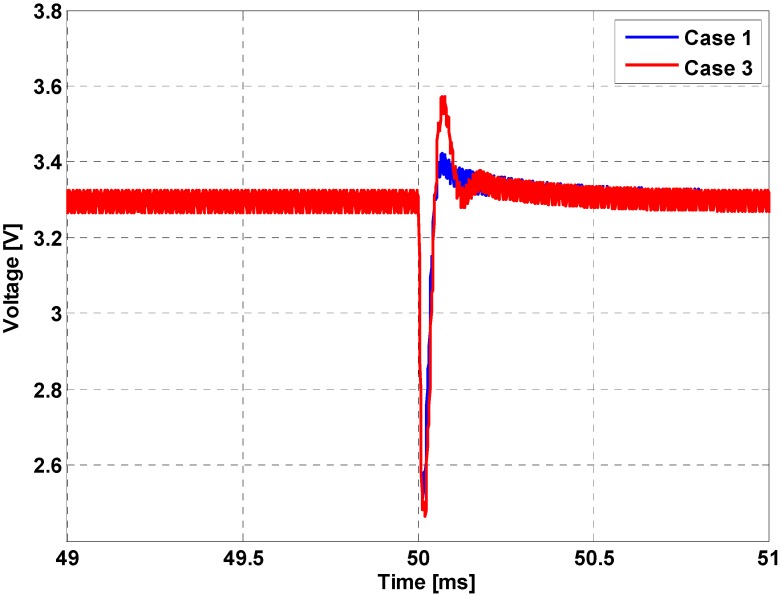
Load regulation performance comparison.

In order to support the analysis of Section 4, a simulation was performed to examine the effects of sensor interface circuit dynamics on the performance of a power circuit. The parameters listed in Table 2 and Table 3 were used for the simulation. Figure 9 shows the load regulation performance. The voltage reference was set to 3.3 V. As shown in Figure 9, the load voltage experiences a short transient period that is cleared within 0.5 ms. The response of Case 3 shows a larger overshoot value compared to that of Case 1. The overshoot of Case 3 (0.3/3.3 = 9.09%) is approximately three times larger than Case 1 (0.1/3.3 = 3.03%). Such an increase in the overshoot value can be explained by the decrease in the PM value, as discussed in the previous section.

**Figure 10:**
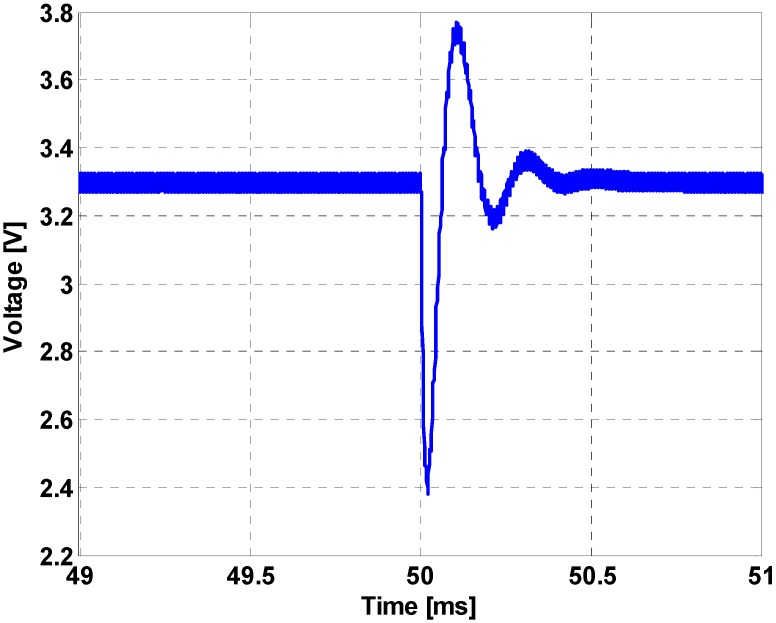
Load regulation performance (20 kHz).

An additional simulation was performed to demonstrate the effects of interface circuits on the regulation performance of power circuits during the transient period, as shown in Figure 10. The low pass filter cut-off frequency was set to 20 kHz, which is five times slower than in Case 3, as shown in Figure 9. Compared to the waveforms of Figure 9, it can be seen that the transient response shown in Figure 10 is more oscillatory and requires more time for the transient responses to be cleared in this case. Such a simulation result shows that the interface circuit should not be ignored during the controller design process.

**Figure 11:**
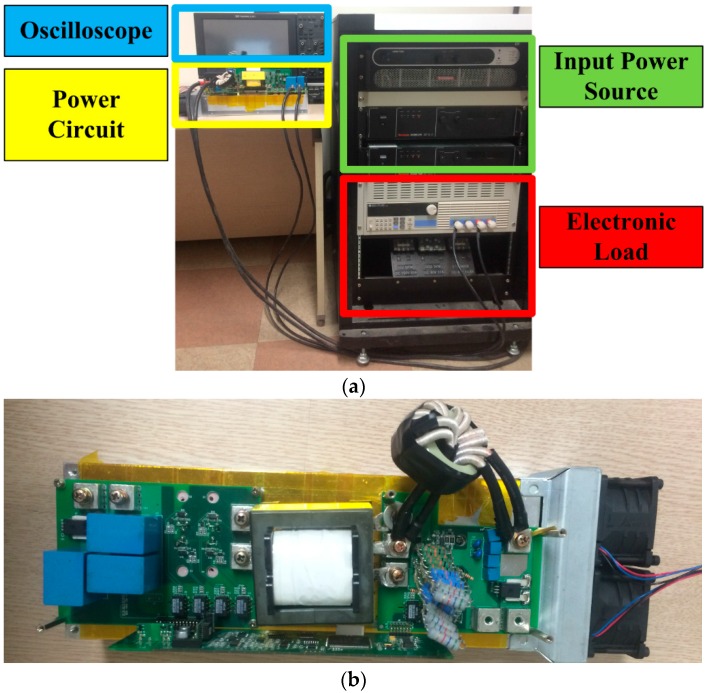
(**a**) Configuration of the experiment setup; (**b**) prototype of a power circuit.

An experiment was also performed to demonstrate that the dynamics of the interface circuits affect the control performance of power circuits. Figure 11 shows the configuration of the experiment setup.

**Figure 12:**
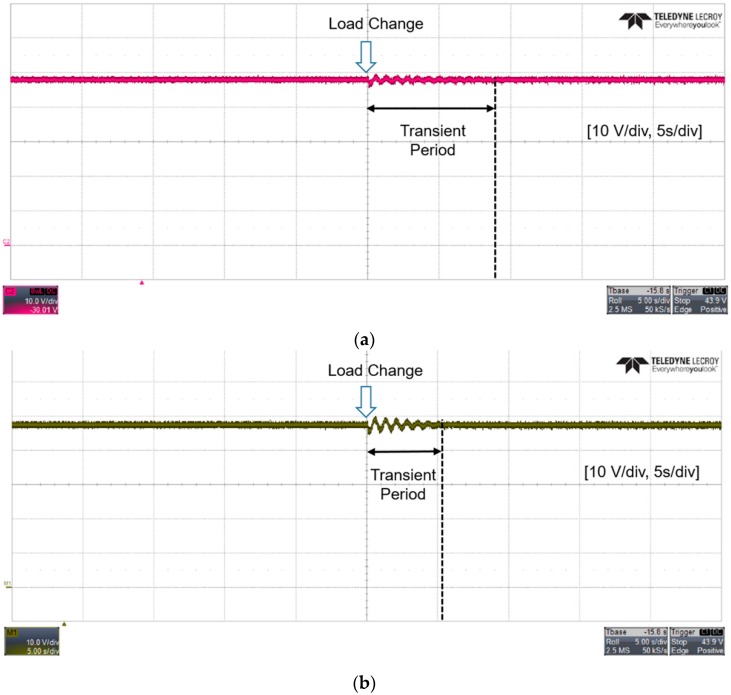
Voltage waveforms with different filter time constants (**a**) 50 ms; (**b**) 0.1 ms.

The step-up operation (i.e., boost mode) of the current-fed push-pull converter was performed. Figure 12 shows that the output voltage waveforms using two different digital low pass filters.

In the experiment, two different filter time constants (i.e., 50 ms and 0.1 ms) were considered, and the input voltage of the experimented power supply interface was 14 V. Throughout the experiment, the output voltage was commanded to be regulated to 48 V. While the voltage showed a satisfactory performance until the load changed, the output voltage showed a transient response period as soon as the load power was changed from 50 W to 150 W. A comparison of Figure 12a,b shows that the dynamics of the interface circuits can affect the transient response characteristics of the power circuits.

## 6. Conclusions

This study examined how the reliability and transient response characteristics of power circuits used in the WoT devices are affected by sensors and interface circuits. As sensors play an essential role in a power circuit control unit, it is necessary to study their potential effects on the overall system reliability and control performance. A quantitative reliability analysis was performed to provide detailed information of the feedback mechanism used in power circuits. The sensor failure rate data were used to characterize how the sensor hardware itself could affect the power circuit reliability. In addition, this paper studied how the system reliability is affected by introducing different types of sensing mechanisms to acquire feedback information such as reconstruction or estimation approaches. The reliability analysis result showed that the overall system reliability depends not only on the failure rate of the sensor, but also on how the system is designed to operate with sensor faults. In particular, it was shown that a higher level of system reliability can be achieved at the cost of installing additional sensors. Furthermore, the analysis result can characterize the reliability performance in a quantitative manner so that further optimization can be conducted during the power circuit design stage to satisfy multiple objectives using a systematic approach. The verification results demonstrated the importance of the sensor being operational by showing the case of power circuits losing regulation capability. In addition, the effects of sensor interface circuits on the stability characteristics were explored by studying the dynamic frequency response of the control loop gain. The analysis results in this paper showed that the control loop phase margin is affected by the dynamics of the sensor interface circuits. The effects of sensor faults and its interface circuit dynamics on the system performance were also verified by both simulations and experiments.

As the proposed analysis approach can be easily applied to other types of power circuits in WoT devices, the analysis approach on reliability and dynamic properties of the sensors, their interface circuits, and alternative sensing approaches is under investigation for future work. Examples of such work include the comprehensive reliability analysis of power circuits for various WoT devices used at different operation conditions (e.g., environment, and operation periods), the development of design standards on sensor integration of power circuits used in WoT devices, and the effects of different reliability models on the estimation accuracy of reliability performance. Exemplar case studies on reliability of actual WoT devices are also works of interest to be performed.

## Figures and Tables

**Table 1:** Cases of the current sensor status.

Case Number	Inductor Current	Capacitor Current	Load Current
1	O	O	O
2	O	X	O
3	O	O	X
4	O	X	X
5	X	O	O
6	X	X	O
7	X	O	X
8	X	X	X

**Table 2:** System parameter values used for interface circuit effects.

System Parameter	Value
Inductance	25 μH
Capacitance	22 μF
Source Voltage	16 V
Voltage Controller	*K_p_* = 0.2, *K_i_* = 1000
Switching Frequency	100 kHz

**Table 3:** Time constant and PM of considered cases.

Case Number	Interface Circuit Time Constant	PM
Case 1	1 μs	63.1°
Case 2	5 μs	51.7°
Case 3	10 μs	42.7°

**Table 4:** System parameters used for sensor failure (Figure 8).

System Parameter	Value
Inductanace	1 mH
Capacitance	125 μF
Source Voltage	50 V
Voltage Controller	*K_p_* = 0.1, *K_i_* = 83.33
Current Controller	*K_p_* = 0.666, *K_i_* = 5555
Switching Frequency	100 kHz

## References

[1] Zou T., Lin S., Feng Q., Chen Y. (2016). Energy-Efficient Control with Harvesting Predictions for Solar-Powered Wireless Sensor Networks. Sensors.

[2] Hwang Y.-S., Chen J.-J., Lai B.-H., Ku Y.-T., Yu C.-C. (2016). A Fast-Transient Boost Converter with Noise-Reduction Techniques for Wireless Sensor Networks. IEEE Sens. J..

[3] Martinez B., Montón M., Vilajosana I., Prades J.D. (2015). The Power of Models: Modeling Power Consumption for IoT Devices. IEEE Sens. J..

[4] Gubbi J., Buyya R., Marusic S., Palaniswami M. (2013). Internet of Things (IoT): A vision, architectural elements, and future directions. Future Gener. Comput. Syst..

[5] Ahn J.-H., Kim D.-H., Lee B.-K., Jin H.-C., Shim J.-S. (2012). DC Appliance Safety Standards Guideline through Comparative Analysis of AC and DC Supplied Home Appliances. J. Electr. Eng. Technol..

[6] Ahmad M. Reliability Models for the Internet of Things: A Paradigm Shift. Proceedings of the 2014 IEEE International Symposium on Software Reliability Engineering Workshops (ISSREW).

[7] Jafer I., Stack P., MacNamee K. (2016). Design of New Power Management Circuit for Light Energy Harvesting System. Sensors.

[8] Brunelli D. (2016). A High-Efficiency Wind Energy Harvester for Autonomous Embedded Systems. Sensors.

[9] Burgos R., Chen G., Wang F., Boroyevich D., Odendaal W.G., Van Wyk J.D. (2012). Reliability-Oriented Design of Three-Phase Power Converters for Aircraft Applications. IEEE Trans. Aerosp. Electron. Syst..

[10] Yu X., Khambadkone A.M. (2012). Reliability Analysis and Cost Optimization of Parallel-Inverter System. IEEE Trans. Ind. Electron..

[11] Kolar J., Krismer F., Lobsiger Y., Muhlethaler J., Nussbaumer T., Minibock J. Extreme Efficiency Power Electronics. Proceedings of the 2012 International Conference on Integrated Power Electronics Systems (CIPS).

[12] Bazzi A.M., Dominguez-Garcia A., Krein P.T. (2012). Markov Reliability Modeling for Induction Motor Drives under Field-Oriented Control. IEEE Trans. Power Electron..

[13] Fang C.-C., Redl R. (2014). Subharmonic Stability Limits for the Buck Converter with Ripple-Based Constant on-Time Control and Feedback Filter. IEEE Trans. Power Electron..

[14] Julian A.L., Oriti G. (2007). A Comparison of Redundant Inverter Topologies to Improve Voltage Source Inverter Reliability. IEEE Trans. Ind. Appl..

[15] Tong Q., Chen C., Zhang Q., Zou X. (2015). A Sensorless Predictive Current Controlled Boost Converter by using and EKF with Load Variation Effect Elimination Function. Sensors.

[16] Midya P., Krein P.T., Greuel M.F. (2001). Sensorless Current Mode Control-An Observer-Based Technique for DC-DC Converters. IEEE Trans. Power Electron..

[17] Kimball J.W., Krein P.T., Chen Y. (2006). Hysteresis and Delta Modulation Control of Converters using Sensorless Current Mode. IEEE Trans. Power Electron..

[18] Lopez V.M., Azcondo F.J., de Castro A., Zane R. (2014). Universal Digital Controller for Boost CCM Power Factor Correction Stages Based on Current Rebuilding Concept. IEEE Trans. Power Electron..

[19] Kim H., Falahi M., Jahns T.M., Degner M.W. (2011). Inductor Current Measurement and Regulation Using a Single DC Link Current Sensor for Interleaved DC-DC Converters. IEEE Trans. Power Electron..

[20] Li Y., Yu H., Su B., Shang Y. (2008). Hybrid Micropower Source for Wireless Sensor Network. IEEE Sens. J..

[21] Abdi B., Ranjbar A.H., Gharehpetian G.B., Milimonfared J. (2009). Reliability Considerations for Parallel Performance of Semiconductor Switches in High-Power Switching Power Supplies. IEEE Trans. Ind. Electron..

[22] Chan F., Calleja H. (2011). Reliability Estimation of Three Single-Phase Topologies in Grid-Connected PV Systems. IEEE Trans. Ind. Electron..

[23] Smater S.S., Dominguez-Garcia A.D. (2011). A Framework for Reliability and Performance Assessment of Wind Energy Conversion Systems. IEEE Trans. Power Syst..

[24] Dhople S.V., Davoudi A., Domínguez-García A.D., Chapman P.L. (2012). A Unified Approach to Reliability Assessment of Multiphase DC–DC Converters in Photovoltaic Energy Conversion Systems. IEEE Trans. Power Electron..

[25] Julian A.L., Oriti G., Blevins S.T. (2010). Operating Standby Redundant Controller to Improve Voltage-Source Inverter Reliability. IEEE Trans. Ind. Appl..

[26] Geyer T., Schroder S. (2010). Reliability Considerations and Fault-Handling Strategies for Multi-MW Modular Drive systems. IEEE Trans. Ind. Appl..

[27] Jiang W., Fahimi B. (2010). Current Reconstruction Techniques for Survivable Three-Phase PWM Converters. IEEE Trans. Power Electron..

[28] Aroudi A.E., Calvente J., Giral R., Al-Numay M., Martinez-Salamero L. (2016). Boundaries of Subharmonic Oscillations Associated to Filtering Effects of Controllers and Current Sensors in Switched Converters Under CMC. IEEE Trans. Ind. Electron..

[29] Chen J.-J., Hwang Y.-S., Yu J.-H., Ku Y.-T., Yu C.-C. (2016). A Low-EMI Buck Converter Suitable for Wireless Sensor Networks with Spur-Reduction Techniques. IEEE Sens. J..

[30] Krug M., Hartmann J., Gröll L., Gengenbach U., Nagel J., Bretthauer G. Model-Based Dual-Mode Controller for Low-Power Buck Converters. Proceedings of the 2014 International Conference on Optimization of Electrical and Electronic Equipment (OPTIM).

[31] Krein P.T. (1998). Elements of Power Electronics.

[32] Hartmann L.V., Corrêa M.B., Lima A.M. A Simple and Effective Control Strategy for Improved Operation of a Current-Fed Push-Pull Converter. Proceedings of the 2010 IEEE Energy Conversion Congress and Exposition.

[33] Tsang K., Chan W. (2005). Cascade Controller for DC/DC Buck Convertor. IEE Proc. Electr. Power Appl..

[34] Dallago E., Passoni M., Sassone G. (2000). Lossless Current Sensing in Low-Voltage High-Current DC/DC Modular Supplies. IEEE Trans. Ind. Electron..

[35] Pajer R., Milanoviĉ M., Premzel B., Rodiĉ M. (2015). MOS-FET as a Current Sensor in Power Electronics Converters. Sensors.

[36] Patel A., Ferdowsi M. (2009). Current Sensing for Automotive Electronics—A Survey. IEEE Trans. Veh. Technol..

[37] LF 2005-S/SP13 User Guide, LEM SA. http://www.lem.com/docs/manuals/LF%202005-S%20SP13_en.pdf.

[38] Kapat S., Krein P.T. (2012). Formulation of PID control for DC–DC converters based on capacitor current: A geometric context. IEEE Trans. Power Electron..

[39] Lukic Z., Zhao Z., Ahsanuzzaman S., Prodic A. Self-Tuning Digital Current Estimator for Low-Power Switching Converters. Proceedings of the 2008 IEEE Applied Power Electronics Conference and Exposition (APEC).

[40] Mezger F., Killat D. A Digital Observer based Current Loop Control for Buck Converters. Proceedings of the 2013 Conference of the IEEE Industrial Electronics Society (IECON).

[41] Castelló J., Espí J.M., García-Gil R. (2016). A New Generalized Robust Predictive Current Control for Grid-Connected Inverters Compensates Anti-Aliasing Filters Delay. IEEE Trans. Ind. Electron..

[42] Ellis G. (2004). Control System Design Guide: A Practical Guide.

[43] Erickson R.W., Maksimovic D. (2001). Fundamentals of Power Electronics.

[44] Chang Y.-T., Lai Y.-S. Effect of Sampling Frequency of A/D Converter on Controller Stability and Bandwidth of Digital-Controlled Power Converter. Proceedings of the 2007 International Conference on Power Electronics (ICPE).

